# Using Bayesian regression to test hypotheses about relationships between parameters and covariates in cognitive models

**DOI:** 10.3758/s13428-017-0940-4

**Published:** 2017-08-25

**Authors:** Udo Boehm, Helen Steingroever, Eric-Jan Wagenmakers

**Affiliations:** 1 Department of Experimental Psychology, University of Groningen, Grote Kruisstraat 2/1, 9712TS Groningen, The Netherlands; 2 Department of Psychology, University of Amsterdam, 1018 XA Amsterdam, The Netherlands

**Keywords:** Bayes factor, Bayesian regression, Computational models, Reinforcement learning models

## Abstract

An important tool in the advancement of cognitive science are quantitative models that represent different cognitive variables in terms of model parameters. To evaluate such models, their parameters are typically tested for relationships with behavioral and physiological variables that are thought to reflect specific cognitive processes. However, many models do not come equipped with the statistical framework needed to relate model parameters to covariates. Instead, researchers often revert to classifying participants into groups depending on their values on the covariates, and subsequently comparing the estimated model parameters between these groups. Here we develop a comprehensive solution to the covariate problem in the form of a Bayesian regression framework. Our framework can be easily added to existing cognitive models and allows researchers to quantify the evidential support for relationships between covariates and model parameters using Bayes factors. Moreover, we present a simulation study that demonstrates the superiority of the Bayesian regression framework to the conventional classification-based approach.

## Introduction

One major motivation for the development of cognitive models is to formalize theories of how latent cognitive variables underlie human behavior. Specifically, model parameters are often used to describe cognitive variables that are related to observed behavior through the model equations. Reinforcement-learning models, for example, have been developed to explain how the outcomes of previous choices influence human decision makers’ future choice behavior (Ahn et al., 2008; Busemeyer & Stout, 2002; Sutton & Barton 1998). Many of these models include a risk-aversion parameter that describes the impact of negative relative to positive outcomes on decision makers’ future choices. If the risk aversion parameter is set to a higher value, these models predict that choice options that yield negative outcomes become much less likely to be chosen in the future whereas choice options that yield positive outcomes only become slightly more likely to be chosen in the future (Steingroever et al. 2013; Ahn et al. 2008).

In recent years there has been increasing interest in explaining individual differences in such model parameters by differences in covariates (e.g., Ahn et al., 2014; Badre et al., 2014; Beitz et al., 2014; Chevalier et al., 2014; Cooper et al., 2015; Kwak et al., 2014; Vassileva et al., 2013). Researchers might, for example, want to test whether a continuous covariate such as a test score or age is related to participants’ estimated risk aversion. However, many models do not come equipped with a principled way of relating covariates to model parameters. The goal of the present work is to develop a hierarchical Bayesian regression framework that allows researchers to estimate and test for relationships between model parameters and covariates.

One strategy researchers have traditionally used to test for relationships between model parameters and covariates is to first group participants according to their values on the covariates, then fit a cognitive model to participants’ behavioral data, and subsequently test the groups of participants for differences in the estimated model parameters (e.g., Vandekerckhove et al., 2011). For instance, Cooper et al. (2015) asked participants to fill out the Regulatory Focus Questionnaire (Higgins et al. 2001) which consists of two scales that measure participants’ tendency to either avoid new tasks for fear of failure (prevention focus) or approach new tasks with an anticipation of success (promotion focus). Cooper et al. categorized participants into two groups based on whether they scored higher on the prevention focus scale or on the promotion focus scale. Subsequently, participants performed 250 trials of one of two versions of the Mars Farming task (Worthy et al. 2011). In the gain-maximization version of the task, participants have to make choices that maximize their total rewards whereas in the loss-minimization version of the task, participants have to make choices that minimize their total losses. In both versions of the task participants have to repeatedly choose between two options. Unbeknownst to participants, the rewards for each choice option depend on their previous choices, with the returns for one option slowly increasing as the option is chosen more often and the returns for the other option decreasing as the option is chosen more often.

Cooper et al. analyzed their data by first fitting a reinforcement-learning model to each individual participant’s choice data and subsequently using ANOVAs to compare the estimated model parameters across groups of participants and task versions. Their main finding was a significant interaction between regulatory-focus group and task version for the model parameter that reflects the degree to which participants use goal-directed behavior. Cooper et al. concluded that a regulatory focus that matches the task structure promotes the use of goal-directed behavior. Although the analysis procedure used by Cooper et al. might seem a reasonable way of testing which covariates are related to which model parameters, it is statistically suboptimal.

Dividing participants into groups based on their scores on a covariate constitutes an artificial dichotomization of a continuous variable, which can lead to biased statistical tests. This problem has been discussed repeatedly in the context of frequentist statistics (Altman & Royston, 2006; Austin & Brunner, 2004; Cohen, 1983; MacCallum et al., 2002; Maxwell & Delaney, 1993; Royston et al., 2006). Despite these repeated warnings, several authors have recently applied dichotomization of continuous covariates to test for relationships with model parameters (e.g., Cooper et al., 2015; Kwak et al., 2014; Steingroever et al., in press). The type of bias introduced by such dichotomization-based tests depends on the correlation between covariates; uncorrelated covariates lead to reduced power (i.e., tests missing true relationships between covariates and model parameters) whereas correlated covariates lead to an inflation of the Type I error rate (i.e., tests detecting spurious relationships between covariates and model parameters). Maxwell & Delaney (1993) provide an accessible explanation of the mechanisms underlying these biases, which we briefly summarize here. As the focus of our work is on the case of continuous, jointly normally distributed model parameters and covariates, a suitable analysis approach is linear regression, which we will use as our comparison standard.

**Fig. 1:**
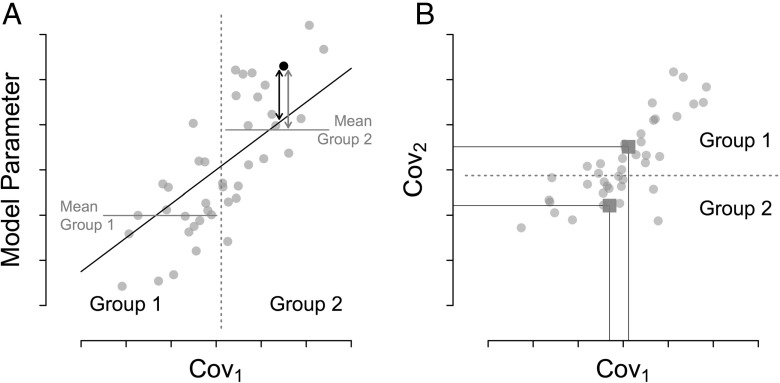
Two biases in analyzing dichotomized variables. **a** Error variance in t-test based on dichotomization compared to regression analysis. The scatterplot shows the relationship between a covariate and a model parameter (*gray dots*), the *dashed line* indicates the median of the covariate, horizontal *black lines* show the mean parameter values for each group obtained by dichotomization along the median, the gray arrow indicates the resulting error for one data point. The *diagonal black line* shows the least-squares regression line, the *black arrow* indicates the associated error. **b** Scatterplot showing the relationship between two correlated covariates (*gray dots*). The *dashed line* indicates the median of covariate 2, and the dark gray squares show the mean value on both covariates of each group obtained by dichotomizing covariate 2 along the median

First, consider a scenario where a researcher measures two uncorrelated continuous covariates, one of which is correlated with a specific model parameter while the other is not. For example, the researcher might administer a questionnaire with two uncorrelated subscales that measure participants’ preference for deliberate and intuitive decision-making, and ask participants to complete 100 trials of a risky decision-making task. The researcher then fits a reinforcement-learning model with a loss-aversion parameter to participants’ choice data. Unbeknownst to the researcher, the loss-aversion parameter is related to participants’ preference for deliberate decision-making but unrelated to their preference for intuitive decision-making. To test for relationships between the model parameter and the covariates, the researcher splits participants’ questionnaire scores on each subscale into two halves based on, say, the median score of each subscale, and, for each subscale, uses a t-test to compare the loss-aversion parameter values of participants scoring below-median (group 1) to the values of participants scoring above-median (group 2). Panel A of Fig. 1 illustrates this scenario for the deliberation scale, which is positively correlated with the loss-aversion parameter. The two horizontal lines show the mean parameter values of each group, the black diagonal line is the result of the correct regression analysis. As can be seen, within each group the deviation of most individual data points from the regression line, that is, the error variance, is much smaller than the deviation from the corresponding group mean. Consequently, a t-test for a difference in group means, which is just the ratio of the mean differences to the error variance, will be biased towards the null hypothesis. A t-test of the regression slope, on the other hand, uses the correct estimate for the error variance and will therefore not show such a bias.

Second, consider a scenario where a researcher measures two correlated continuous covariates, one of which is correlated with a specific model parameter while the other is not. In our previous example, the deliberate decision-making subscale and the intuitive decision-making subscale might be correlated with each other, and the loss-aversion model parameter might be correlated with the deliberate decision-making subscale but not with the intuitive decision-making subscale. To test for relationships between the model parameter and the covariates, the researcher again splits each subscale into two halves and, for each subscale, uses a t-test to compare the parameter values of participants scoring below-median (group 1) to the values of participants scoring above-median (group 2). In this case the covariate of interest is the intuition subscale, which is not correlated with the loss-aversion parameter. Panel B of Fig. 1 shows a scatterplot of participants’ scores on the two subscales with the deliberation scale on the x-axis and the intuition scale on the y-axis; the dark gray squares indicate the means of both subscales for each group created by splitting the deliberation scale into two halves. As can be seen, the mean value on the intuition scale, which is not correlated with the loss-aversion parameter, is higher for one group than for the other. However, because the two subscales are correlated, the two groups also differ in their mean on the deliberation scale, which is correlated with the loss-aversion parameter. Therefore, a t-test for a mean difference in the model parameter between the two groups might suggest a relationship between the intuition scale and the model parameter due to the difference in means on the deliberation scale. A regression analysis, on the other hand, avoids this problem because it partials out the correlation between the two covariates before relating the intuition scale to the model parameter. It should be clear from the above examples that dichotomization of continuous covariates is a problematic practice and the associated biases can be easily avoided by using an appropriate regression analysis.

As mentioned above, the problem of dichotomization-based analyses has been discussed previously in the context of frequentist statistical testing and a potential solution is offered by maximum-likelihood based regression extensions that are available for some cognitive models (e.g., Coolin et al., 2015). However, a discussion of the effects of dichotomization on Bayesian hypothesis testing and corresponding solutions in the form of a Bayesian regression framework for hypothesis testing in cognitive models are missing. This is a particularly pertinent issue as hierarchical Bayesian models have been gaining popularity in recent years (e.g., Lee, 2011; Rouder and Lu, 2005; Rouder et al., 2003). Although hierarchical Bayesian regression extensions have been developed for some cognitive models, the focus of this work has mostly been on parameter estimation rather than hypothesis testing. For example, Heck et al. (in press) present an R package for fitting hierarchical Bayesian multinomial processing tree models. Their package includes, among other features, a regression extension that allows researchers to add covariates as predictors for models parameters. However, Heck et al. do not discuss the problem of Bayesian hypothesis testing for relationships between model parameters and covariates. Moreover, Heck et al.’s implementation is based on the assumption that covariates are uncorrelated, which is not the case for many covariates of practical interest, such as clinical test scores and personality inventories (e.g., Anderson et al., 2015; King & Jackson, 2009).

The goal of the present work is to develop a regression framework for hierarchical Bayesian cognitive models that allows researchers to directly test for relationships between model parameters and covariates. In the following sections we will first introduce a reinforcement-learning model that will serve as an example application. We will then give a short overview of hypothesis testing in the context of Bayesian regression models before we develop our hierarchical Bayesian regression extension for cognitive models. Finally, we will present a simulation study in which we compare the effects of regression-based and dichotomization-based analyses on Bayesian hypothesis testing.

## Regression framework for relating cognitive model parameters to covariates

In this section we will develop a regression framework for relating model parameters to covariates. As an illustrative example, we will apply our regression framework to a popular reinforcement-learning model, the Prospect Valence Learning model with the delta learning rule (PVL-Delta; (Ahn et al. 2008; Fridberg et al. 2010; Steingroever et al. 2013; 2014)). Nevertheless, our regression framework can easily be applied to different reinforcement-learning models (Busemeyer & Stout, 2002; Sutton & Barton, 1998) as well as other types of cognitive models such as multinomial processing trees (Batchelder & Riefer 1999; Coolin et al. 2015; Matzke et al. 2015; Riefer & Batchelder 1988) or sequential sampling models (Brown & Heathcote 2008; van Ravenzwaaij et al. in press).

The PVL-Delta model was developed to disentangle the psychological processes driving risky decision-making in the Iowa-gambling task (IGT; Bechara et al., 1994). We will first briefly outline the structure of the IGT and give a short summary of the PVL-Delta model and its hierarchical Bayesian implementation before we develop our regression framework.

### Iowa gambling task and hierarchical PVL-delta model

The IGT is an economic decision-making task that aims to measure decision-making deficits in clinical populations. In the computerized version of the IGT, participants are given an initial credit of $2000 and are presented with four decks of cards, each of which is associated with a characteristic payoff structure. On each trial, participants pick a card and receive feedback about the wins and losses for that card, as well as the running tally. Participants are instructed to choose cards from the decks in a way that maximizes their long-term net outcomes (see Bechara et al., 1994 for more details on the task).

The PVL-Delta model formalizes assumption about the cognitive processes by which participants learn to optimize their behavior in risky decision-making and, specifically, how to maximize their long-term net outcomes on the IGT. The model conceptualizes risky decision-making as a three-step process. On each trial *t* = 1,…,*T* a participant chooses a card from deck *k* ∈{1,2,3,4} and evaluates the net outcome *X*(*t*) of the current decision using a non-linear utility function. This so-called prospect utility function (Tversky & Kahneman 1992) is governed by two parameters:

1 $$ u_{k}(t)= \left\{\begin{array}{ll} X(t)^{A} & \quad \text{ if } X(t) \geq 0\\ -w \cdot |X(t)|^{A} & \quad \text{ if } X(t) < 0,\\ \end{array}\right.  $$

where *A* ∈ [0,1] is the outcome sensitivity parameter, and *w* ∈ [0,5] is the loss aversion parameter. The outcome sensitivity parameter determines the shape of the utility function. As *A* approaches 1, the utility function becomes more linear, meaning that the subjective utility of the decks increases proportionally with increasing net outcomes, whereas as *A* approaches 0, the utility function approximates a step function, meaning that the subjective utility is determined only by the sign of the net outcomes but not their actual value. The loss aversion parameter determines the impact of negative net outcomes; a value of *w* close to 0 means that negative net outcomes are neglected, a value of 1 indicates an equal impact of negative and positive net outcomes on the subjective utility, and a value closer to 5 indicates a large impact of negative net outcomes.

In a second step the model assumes that the participant updates the expected utility of the chosen deck based on the subjective utility of the current trial. This updating process is governed by the so-called delta-learning rule (Rescorla & Wagner 1972):

2 $$ Ev_{k}(t) = Ev_{k}(t-1) + a \cdot (u_{k}(t)-Ev_{k}(t-1)),  $$

where *E* *v*_*k*_(*t*) is the expected utility on trial *t*, and *a* ∈ [0,1] is the updating parameter that determines how past expectancies influence the evaluation of the current outcome. A value of *a* close to 1 indicates quick forgetting and strong recency effects while a value of *a* close to 0 indicates slow forgetting and weak recency effects.

In a third step the participant makes a new decision based on the expected utilities. The choice process is governed by the softmax rule (Luce 1959):

3 $$ P[S_{k}(t+1)]=\frac{e^{\theta \cdot Ev_{k}(t)}}{{\sum}_{j=1}^{4} e^{\theta \cdot Ev_{j}(t)}},  $$

where *P*[*S*_*k*_(*t* + 1)] is the probability of choosing deck *k* on the (*t* + 1)th trial, and *𝜃* is a sensitivity parameter that controls how closely choice probabilities match the expected deck utilities. A value of *𝜃* close to 0 indicates random choice behavior while larger values indicate choice behavior that matches the expected utilities more closely. The sensitivity parameter, in turn, is determined by

4 $$ \theta=3^{c}-1,  $$

where *c* ∈ [0,5] is the consistency parameter that determines the relative amount of exploitation vs exploration; values of *c* close to 0 cause random choice behavior whereas larger values cause more deterministic behavior.

**Fig. 2:**
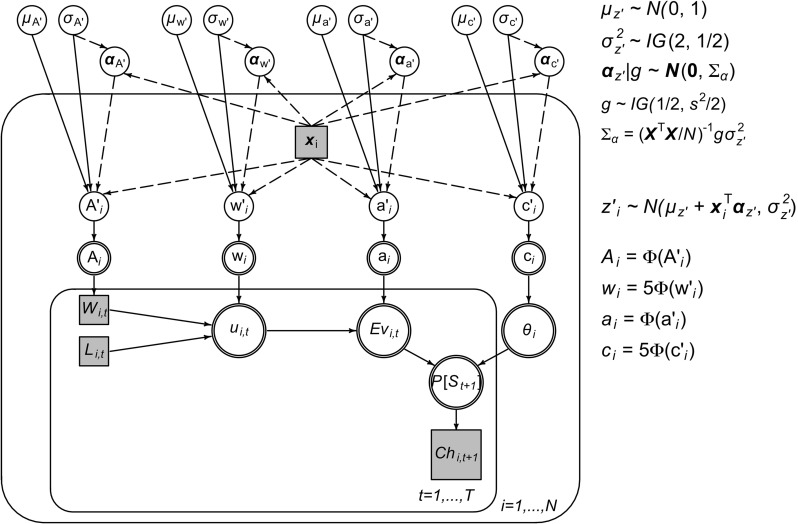
Hierarchical PVL-Delta model with regression extension. Solid edges indicate components of Steingroever et al.’s (in press) hierarchical implementation of the PVL-Delta model; newly added regression components for relating model parameters to covariates are indicated by dashed edges. *z* *i*′ denotes the probit-transform of model parameter *z*_*i*_ ∈{*A*_*i*_,*w*_*i*_,*a*_*i*_,*c*_*i*_}

Steingroever et al. (in press) have presented a Bayesian hierarchical implementation of the PVL-Delta model (solid edges in Fig. 2; see also Steingroever et al., 2013, 2014; Wetzels et al., 2010), for a hierarchical implementation of the related “EV” model). In their implementation of the model, trials *t* of the IGT (inner plate) are nested within participants *i* (outer plate). For each trial *t* of participant *i* the choice of a deck of cards on the subsequent trial *C* *h*_*i*,*t*+1_, and the wins *W*_*i*,*t*_ and losses *L*_*i*,*t*_ on the current trial are observed nodes (gray rectangles); the utility *U*_*k*,*i*,*t*_, expected utility *E* *v*_*k*,*i*,*t*_, sensitivity parameter *𝜃*_*i*_, and probability of choosing deck *k* on the next trial *P*[*S*_*k*,*i*_(*t* + 1)] are deterministic nodes (double-bordered circles; note that we dropped the subscript *k* in the graphical model for improved readability) as they are fully determined by the model equations and parameters. Moreover, the individual-level model parameters *z*_*i*_ ∈{*A*_*i*_,*w*_*i*_,*a*_*i*_,*c*_*i*_} are modeled based on their probit-transforms, which means that the model parameters are treated as deterministic nodes. The probit-transform $z^{\prime }_{i}$ of parameter *z*_*i*_ is $z^{\prime }_{i} = {\Phi }^{-1}(z_{i})$, where Φ^−1^ denotes the inverse of the cumulative distribution function of the standard normal distribution. The probit-transform is a stochastic node (single-bordered circle) sampled from a group-level normal distribution with mean $\mu _{z^{\prime }}$ and standard deviation $\sigma _{z^{\prime }}$. The priors for the group-level parameters are independent standard normal distributions for the group-level means, $\mu _{z^{\prime }} \sim \mathscr {N}(0,1)$, where $ \mathscr {N}(\mu , \sigma ^{2})$ denotes the normal distribution with mean *μ* and variance *σ* ^2^, and uniform distributions for the group-level standard deviations, $\sigma _{z^{\prime }} \sim \mathscr {U}(0,1.5)$, where $\mathscr {U}(a, b)$ is the uniform distribution ranging from *a* to *b*.

### Regression in statistical models

Bayesian regression methods have been largely developed in the context of statistical models (Jeffreys 1961; Liang et al. 2008; Rouder & Morey 2012). In this section we will review relevant results from the statistical literature before we adapt them for our example model in the next section.

Hypothesis testing in the context of regression is a model selection process. Given a set of predictors, the goal is to select a subset that best accounts for the observed data. Consider, for example, the simple situation where a researcher has a criterion variable *y* and a single predictor variable *x* with mean 0 and variance 1 and wants to know whether *x* has any predictive value for *y*. To answer this question, the researcher constructs two models. The null model, $\mathscr {M}_{0}$, only includes an intercept term *μ* and assumes that the values of *y* are normally distributed around this intercept:

5 $$ y \sim \mathscr{N}(\mu, \sigma^{2}),  $$

where *σ* ^2^ is the residual variance of the criterion variable. The alternative model, $\mathscr {M}_{1}$, additionally includes the predictor variable *x*:

6 $$ y \sim \mathscr{N}(\mu + \alpha_{x} x, \sigma^{2}),  $$

where *α*_*x*_ is a regression weight and *σ* ^2^ is again the residual variance. The researcher then compares the adequacy of the two models for the data at hand and selects the corresponding model.

In a more general setting, a researcher might consider a set of predictor variables *x*_.*j*_, *j* = 1…*P* with observations *x*_*ij*_, *i* = 1…*N* for each predictor. We again assume that each predictor has mean 0. The researcher now wants to select the subset of predictors that are related to the criterion variable *y*_*i*_. The full model relating all predictors to the criterion variable then is:

7 $$ y_{i} \sim \mathscr{N}(\mu + \alpha_{1} x_{i1} + \alpha_{2} x_{i2} \hdots \alpha_{P} x_{iP}, \sigma^{2}),  $$

where *α*_*j*_ is a regression weight for predictor *j* and *σ* ^2^ is the residual variance. This model can be more conveniently expressed in vector notation:

8 $$ y_{i} \sim \mathscr{N}(\mu + \pmb{x_{i}}^{T} \pmb{\alpha}, \sigma^{2}).  $$

Here *α* = [*α*_1_,…,*α*_*P*_] denotes the *P* × 1 vector of regression weights, and superscript *T* indicates the transpose. We furthermore assume that the predictor variables have mean 0. This can be achieved by centering the predictor, that is, subtracting the mean of the predictor from each observation. The resulting the *P* × 1 vector of predictors for each observation *i* is $\pmb {x}_{i.} = [x_{i1} - \bar {x}_{.1}, \hdots , x_{iP} - \bar {x}_{.P}]$, where $\bar {x}_{.j}$ is the mean across observations of predictor *j*. The researcher can now construct a model that only includes a subset of the predictor variables and test the hypothesis that the reduced model is more adequate for the data than the full model.

Within Bayesian statistics, the principled way of testing such hypotheses is by computing Bayes factors, that is the ratio of the marginal likelihood of the observed data under two competing models, $\text {BF}_{10} = p(y \mid \mathscr {M}_{1})/p(y \mid \mathscr {M}_{0})$ (Berger 2006; Jeffreys 1961; Lewis & Raftery, 1997). Bayes factors hold a number of advantages over conventional tests of statistical significance, as practiced in psychology (Gigerenzer et al. 2004). Firstly, significance tests can only ever reject but never accept the null hypothesis. Bayes factors, on the other hand, can express support for the null hypothesis as well as the alternative hypothesis (Rouder et al. 2009). Secondly, while significance tests force a binary choice upon researchers between rejecting the null hypothesis or remaining in a state of suspended disbelief, Bayes factors allow researchers a graded expression of the evidence for the competing hypotheses provided by their data.^1^ Thirdly, conventional significance tests require researchers to commit to a sampling plan before data collection begins and to continue collecting data even if a hypothesis can be confidently rejected or accepted before the full sample has been acquired. Bayes factors, on the other hand, allow researchers to assess the support for competing hypotheses repeatedly during the sampling process and stop collecting data when a hypothesis is supported or rejected to a satisfying degree (Edwards et al. 1963; Kass & Raftery 1995; Rouder 2014).

Default Bayes factors need to fulfill a number of theoretical desiderata (Bayarri et al. 2012; Rouder & Morey 2012). Firstly, Bayes factors should be location and scale invariant. In the case of regression models, this means that the scale on which the criterion and predictor variables are measured (e.g., kilograms, grams, milligrams) and the location of the zero-point of the scale (e.g., temperature in Celsius vs. in Fahrenheit) should not influence the Bayes factor. Secondly, Bayes factors should be consistent, which means that as sample size approaches infinity, the Bayes factor should converge to the correct bound (i.e., BF_10_ → 0 if $\mathscr {M}_{0}$ is correct and BF_10_ →*∞* if $\mathscr {M}_{1}$ is correct). Thirdly, Bayes factors should be consistent in information, which means that the Bayes factor should not approach a finite value as the information provided by the data in favor of the alternative model approaches infinity. In the case of regression models this means that, as the coefficient of determination, *R* ^2^, in $\mathscr {M}_{1}$ approaches 1, the Bayes factor should go to infinity (Ly et al. 2016).

Whether or not Bayes factors satisfy the above desiderata critically depends on the choice of the priors for the model parameters. Assigning improper priors to model-specific parameters, for instance, leads to indeterminate Bayes factors (Jeffreys 1961). In our example with a single predictor *x*, the corresponding regression weight *α*_*x*_ is included in $\mathscr {M}_{1}$ but not in $\mathscr {M}_{0}$. If *α*_*x*_ is assigned an improper prior that is only determined up to a multiplicative constant, this constant will appear in the numerator of the Bayes factor but not in the denominator, which means that it will not cancel and the Bayes factor BF_10_ will depend on the multiplicative constant. Consequently, researchers need to choose the prior distribution for the model parameters in such a way that model comparisons yield Bayes factors with the desired theoretical properties.

An additional criterion in selecting priors for the model parameters is the degree to which priors are noninformative. In many situations, researchers have little information about the range in which the model parameters, that is, the regression weights, should fall. Therefore, the weights should be assigned a prior that puts little constraint on the possible values. One prior that has regularly been used in regression problems is Zellner’s g-prior (Zellner 1986). In the case of *P* predictor variables and *N* observations for each variable, this prior takes the form:

$$\pmb{\alpha} \mid g \sim \pmb{\mathscr{N}}(\textbf{0},g \sigma^{2} (\pmb{X}^{T} \pmb{X})^{-1}), $$

where *α* is the vector of regression weights, *g* is a scaling factor, *σ* ^2^ is the residual variance of the criterion variable, **0** is a *P* × 1 vector of zeros, and *X* is the *N* × *P* centered design matrix that is obtained by writing the *P* × 1 vector of predictor values for all *N* observations as rows of a matrix: *X* = [*x*_1._,…,*x*_*N*._]^*T*^. $\pmb {\mathscr {N}}(\pmb {\mu }, {\Sigma })$ denotes the multivariate normal distribution with mean vector *μ* and covariance matrix Σ. The degree to which this prior is informative is controlled by its variance-covariance matrix, which in turn depends on *g*, *σ* ^2^, and *X*. In Zellner’s (1986) conceptualization of this prior, the design matrix should be treated as a constant; the prior can then be interpreted as the prior on the regression weights arising from a repetition of the experiment with the same design matrix. The intercept *μ* and the residual variance *σ* ^2^ should be assigned a scale-invariant Jeffreys prior (Jeffreys 1961) *p*(*μ*,*σ* ^2^) ∝ 1/*σ* ^2^. Finally, the scaling factor *g* controls the weight given to the prior relative to the data. For example, *g* = 10 means that the data are given 10 times as much weight as the prior. The scaling factor thus controls how peaked or how informative the prior is.

Another way to understand the effect of the scaling parameter is to consider the shrinkage factor *g*/(1 + *g*) (Liang et al. 2008; Wetzels et al. 2012). Using this shrinkage factor, the posterior mean for *α* can be estimated as the product of the shrinkage factor and the least-squares estimate of the regression weights, *α*_*OLS*_. Consequently, if *g* is set to a small value, the posterior estimate of *α* will be pulled towards 0 whereas a high value of *g* leads to a posterior mean that is similar to the least-squares estimate.

The question that remains is how *g* should be set. One popular choice is to set *g* = *N*, which yields a unit information prior (Kass & Raftery 1995). Specifically, the term *σ* ^2^(*X* ^*T*^ *X*)^−1^ in the expression for the variance-covariance matrix of the prior equals the variance-covariance matrix of the maximum-likelihood estimators of the regression weights, var(*α*_*OLS*_). This estimate is based on the design matrix with *N* rows, which conveys the information of *N* observations. Therefore, by setting *g* to *N*, the influence of the design matrix on the prior can be made equivalent to the information contained in a single observation.^2^

However, as shown by Liang et al. (2008), the Zellner prior in its general form suffers from two shortcomings. Firstly, if *g* is set to a fixed value, the resulting Bayes factors will suffer from the “information paradox”. This means that when a model $\mathscr {M}_{1}$ is compared to the null model $\mathscr {M}_{0}$, and the coefficient of determination *R* ^2^ under $\mathscr {M}_{1}$ approaches 1 (i.e., there is infinite support for $\mathscr {M}_{1}$), the Bayes factor will tend to a finite value, thus violating the theoretical desideratum of consistency in information. Secondly, if *g* is set to a very large value to make the prior noninformative, Bayes factors will suffer from the Jeffreys-Lindley-Bartlett paradox. This means that $\mathscr {M}_{0}$ will unduly be favored. In the limiting case when *g* →*∞*, the Bayes factor will go to zero, irrespective of the information provided by the data, thus violating the theoretical property of consistency.

The problems of the Zellner prior are resolved by the Jeffreys-Zellner-Siow prior (JZS prior; Nuijten et al., 2015; Rouder & Morey, 2012). In the approach suggested by Zellner & Siow (1980), the regression coefficients are assigned a multivariate Cauchy prior, which satisfies the consistency requirements on the Bayes factors (Liang et al. 2008; Wetzels et al. 2012):

9 $$ \pmb{\alpha} \sim \pmb{\mathscr{C}}(\textbf{0}, \sigma^{2} (\pmb{X}^{T} \pmb{X}/N)^{-1} s^{2}),  $$

where $\pmb {\mathscr {C}}(\pmb {\mu }, {\Sigma })$ denotes the multivariate Cauchy distribution with mean vector *μ* = **0** and scale matrix Σ = *σ* ^2^(*X* ^*T*^ *X*/*N*)^−1^, and *s* is a scale parameter that scales the prior to the researcher’s a priori expectations for the vector of regression weights *α*. However, one slight drawback of the multivariate Cauchy prior is that the marginal likelihood of the data under a model cannot be computed in closed form and numerical approximations require the computation of a *P*-dimensional integral, which becomes unstable for models with large numbers of predictors. As pointed out by Liang et al. (2008), a remedy to this problem is to express the multivariate Cauchy distribution as a mixture of g-priors. In this approach, the scaling factor *g* in the Zellner prior is treated as a random variable:

10 $$ \pmb{\alpha} \mid g \sim \pmb{\mathscr{N}}(\textbf{0},g \sigma^{2} (\pmb{X}^{T} \pmb{X}/N)^{-1}),  $$

and *g* is assigned an inverse-gamma prior:

11 $$ g \sim \mathscr{I}\mathscr{G}(1/2,s^{2}/2)  $$

with shape parameter 1/2 and scale parameter *s* ^2^/2. Note that the scale parameter *s* of the inverse gamma distribution is equal to the scale parameter of the multivariate Cauchy distribution. Using this mixture representation of the JZS prior reduces the computation of a Bayes factor to a one-dimensional integral that can be computed numerically with great precision (Rouder & Morey 2012).

The above discussion shows that using the JZS prior yields Bayes factors that are consistent and consistent in information. The resulting Bayes factors are also location and scale-invariant, which can be shown by considering three equivalent methods for obtaining location and scale-invariance. Firstly, in the case of a single predictor *x*, location-invariance of the Bayes factor can be achieved by centering the predictor, and scale-invariance is achieved by standardizing the predictor with respect to the residual standard deviation of the criterion variable, *σ*, and the standard deviation of the predictor, *s*_*x*_:

$$\tilde{x}_{i}=\frac{x_{i}-\bar{x}}{s_{x}}\sigma. $$

Using this standardized predictor in the regression model yields location and scale-invariant Bayes factors. Secondly, an equivalent way to achieve location and scale-invariant Bayes factors is to standardize the regression weight for the centered predictor with respect to the residual standard deviation of the criterion variable and the standard deviation of the predictor. This yields a standardized effect size *β*_*x*_:

12 $$ \beta_{x} = \alpha_{x} \frac{s_{x}}{\sigma},  $$

which can be assigned a univariate Cauchy prior with scale parameter s:

13 $$ \beta_{x} \sim \mathscr{C}(s).  $$

Here *s* describes the interquartile range of plausible values for the standardized effect size *β*_*x*_. Finally, scale-invariance can equivalently be obtained by including the standardization with respect to *σ* and *s*_*x*_ in the prior distribution of the regression weights:

14 $$ \alpha_{x} \sim \mathscr{C}\left( s \frac{\sigma^{2}}{{s^{2}_{x}}}\right).  $$

In the case of multiple predictors, a location and scale-invariant prior can be placed on the vector of regression weights, *α*. In this case standardization is achieved by the term *σ* ^2^(*X* ^*T*^ *X*/*N*)^−1^ in expression for the prior distribution (Eqs. 9 and 10).

### Regression in cognitive models

Our regression framework for cognitive models is based on the Bayesian regression framework presented above. To incorporate the regression framework into Steingroever et al.’s model (in press), we replaced the group-level normal priors for the probit-transformed model parameters by a regression equation that relates the covariates *j* = 1…*P* to the individual-level model parameters $z^{\prime }_{i}$. Specifically, each probit-transformed model parameter for each participant is sampled from a normal distribution whose mean depends on the vector of centered covariates *x*_*i*_:

15 $$ z^{\prime}_{i} \sim \mathscr{N}(\mu_{z^{\prime}} + \pmb{x}^{T}_{i} \pmb{\alpha}_{z^{\prime}}, \sigma^{2}_{z^{\prime}}).  $$

Here $\mu _{z^{\prime }}$ is the intercept term, $\pmb {x}^{T}_{i}$ is a transposed *P* × 1 vector of *P* centered covariate values for participant *i*, $\pmb {\alpha }_{z^{\prime }}$ is the *P* × 1 vector of regression weights for model parameter *z* ^′^, and $\sigma ^{2}_{z^{\prime }}$ is the residual variance of the model parameter *z* ^′^. The standardized effect size for covariate *j* is again a transformation of the conventional regression weight:

16 $$ \beta_{z^{\prime}j} = \alpha_{z^{\prime}j} \frac{s_{j}}{\sigma_{z^{\prime}}},  $$

where $\beta _{z^{\prime }j}$ is the standardized effect size for the regression of parameter *z* ^′^ on covariate *j*, and *s*_*j*_ is the standard deviation of the covariate.

We again retain the three desiderata for Bayes factors by placing a multivariate Cauchy prior on the vector of regression weights $\pmb {\alpha }_{z^{\prime }}$ for each model parameter, which we express as a mixture of g (Zellner & Siow, 1980):

17 $$ \pmb{\alpha}_{z^{\prime}} \mid g \sim \pmb{\mathscr{N}}(\textbf{0},g \sigma^{2}_{z^{\prime}} (\pmb{X}^{T} \pmb{X}/N)^{-1}),  $$

18 $$ g \sim \mathscr{I}\mathscr{G}(1/2,s^{2}/2),  $$

where the scale parameter *s* describes the interquartile range of plausible values for *β*.

Figure 2 shows the graphical implementation of the PVL-Delta model with our regression extension. The model components we added to the hierarchical PVL-Delta model are indicated by dashed edges. As in the hierarchical PVL-Delta model, the probit-transformed model parameters are stochastic nodes that are nested within participants. The model parameters depend on the group-level stochastic nodes $\mu _{z^{\prime }}$, $\sigma _{z^{\prime }}$ and the vector $\pmb {\alpha }_{z^{\prime }}$, as well as the observed vector of covariate values *x*_*i*_ that is nested within participants; the relationship between these quantities is given by the regression Eq. 15. Moreover, the vector $\pmb {\alpha }_{z^{\prime }}$ depends on the vector of covariate values *x*_*i*_ via Eq. 17. Similar to Steingroever et al.’s (2013, in press) implementation of the hierarchical PVL-Delta model, who assigned the group-level mean parameters a standard normal prior, we assigned the intercept $\mu _{z^{\prime }}$ a standard normal prior $\mu _{z^{\prime }} \sim \mathscr {N}(0,1)$. However, instead of the uniform prior used in the hierarchical PVL-Delta model, we assigned the residual variance $\sigma ^{2}_{z^{\prime }}$ an inverse-gamma prior $\sigma ^{2}_{z^{\prime }} \sim \mathscr {I}\mathscr {G}(2,1/2)$ with shape parameter 2 and scale parameter 1/2. Our choice of a relatively informative prior was mainly made to speed up model convergence (see below) and a uniform prior did not yield qualitatively different results. Finally, we assigned the vector of regression weights $\pmb {\alpha }_{z^{\prime }}$ the mixture of g prior described in Eqs. 17–18 and set the scale parameter *s* = 1. The Stan-code for the model can be found at osf.io/6tfz3.

### Computing Bayes factors

Within the regression framework presented above, researchers can test for a relationship between a normally distributed model parameter, in our case the probit-transformed parameter *z* ^′^, and a covariate *x*_*j*_ by computing the Bayes factor for the standardized effect size $\beta _{z^{\prime }j}$. Bayes factors express the relative likelihood of the observed data *y* under two competing hypotheses, $\mathscr {H}_{0}$ and $\mathscr {H}_{1}$ (e.g., Jeffreys, 1961; Rouder et al., 2009):

19 $$ \text{BF}_{10} = \frac{p(y \mid \mathscr{H}_{1})}{p(y \mid \mathscr{H}_{0})}. $$

A sensible null hypothesis might be that the standardized effect size for a specific model parameter *z* ^′^ on the covariate *x*_*j*_ is 0, $\mathscr {H}_{0}: \beta _{z^{\prime }j}=0$, and the alternative hypothesis might state that the effect size is not 0. The exact expression for the alternative hypothesis depends on the marginal prior for standardized effect size under consideration, which in our case is a univariate Cauchy distribution with scale parameter *s* = 1, thus $\mathscr {H}_{1}: \beta _{z^{\prime }j} \sim \mathscr {C}(1)$. In the case of a point-null hypothesis that is nested under the alternative hypothesis, the Bayes factor for the parameter in question can conveniently be obtained as the ratio of the alternative hypothesis’ prior density over its posterior density at the point-null $\text {BF}_{10}=p(\beta _{z^{\prime }j} = 0 \mid \mathscr {H}_{1}) / p(\beta _{z^{\prime }j} = 0 \mid y, \mathscr {H}_{1})$, which is known as the Savage-Dickey density ratio (Dickey & Lientz 1970; Wagenmakers et al. 2010). Note that the Savage-Dickey density ratio can also be used to test more complex null hypotheses involving several effect sizes simultaneously. However, such hypothesis tests will require estimating the multivariate posterior density for the effect sizes involved, which can be challenging in practical applications. In these cases alternative methods for computing Bayes factors, such as bridge sampling (e.g., Gronau et al., 2017), might offer a simpler solution.

## Simulation study

The goal of our simulation study is to demonstrate how dichotomizing continuous covariates biases Bayes factors and how these biases can be avoided using the regression framework developed above. To generate realistic data for our simulations, we first fitted the PVL-Delta model with the regression extension to a published data set (Steingroever et al. in press). We subsequently used the resulting parameter estimates to generate synthetic data for two scenarios, one in which covariates are not correlated with each other, and one in which covariates are correlated. To emulate a typical dichotomization-based analysis strategy, we applied the hierarchical Bayesian PVL-Delta model in combination with a median-split of the covariates to the simulated data. In a median-split analysis, participants are divided into two groups based on whether their value on the covariate lies above or below the median. Finally, we compared the resulting Bayes factors from the dichotomization-based analysis to the Bayes factors obtained from the PVL-Delta model with our regression extension.

### Generating synthetic data

#### Data set

We based the setup for our simulated data on the data published in Steingroever et al. (in press) because of the simple experimental design and the clear structure of the covariates measured. In Steingroever et al.’s study 70 participants performed 100 trials of the IGT using the traditional payoff scheme suggested by Bechara et al. (1994). In addition, they completed Betsch and Ianello’s (2010) decision style questionnaire, which consists of 70 items that assess participants’ tendency to use an intuitive or deliberate decision style on a seven-point Likert scale. Steingroever et al. submitted participants’ responses to a principal component analysis and computed participants’ scores on two factors, deliberation and intuition. In addition, they fitted the PVL-Delta model to participants’ performance data on the IGT and related each participant’s factor scores to the estimated PVL-Delta parameters. A full description of the sample, IGT, and questionnaire data can be found in Steingroever et al. (in press).

We fitted the PVL-Delta model with the regression extension to Steingroever et al.’s IGT data and used participants’ scores on the Deliberation and Intuition scales as covariates *x*_1_ and *x*_2_, respectively. In contrast to Steingroever et al., whose analysis only included the data of participants who scored high on one scale and low on the other, we included the data of all participants in our analysis. As Steingroever et al. reported relatively small effects of the covariates on the model parameters, we expected to also find relatively small standardized effect sizes $\alpha _{z^{\prime }j}$ and therefore set the scale parameter of the Cauchy prior to *s* = 1/3 (Eq. 18).^3^ To fit the PVL-Delta model to the data, we implemented the model with the regression extension in Stan (Carpenter et al., in in press; Stan Development Team, 2016a, b) and obtained samples from the posterior distributions of the model parameters. For each model parameter we ran three MCMC chains and collected 50,000 posterior samples per chain. We discarded the first 5,000 samples of each chain as burn-in samples and furthermore thinned each chain, retaining every fifth sample. Starting values for the population means $\mu _{z^{\prime }}$ were randomly drawn from standard normal distributions, starting values for the population standard deviations $\sigma _{z^{\prime }}$ were randomly drawn from exponential distributions with scale parameter 1, and starting values for the regression weights $\alpha _{z^{\prime }}$ were randomly drawn from normal distributions with mean 0 and standard deviation 2. All chains showed good convergence (Gelman-Rubin diagnostic $\hat {R} \leq 1.005$, Gelman & Rubin, 1992).

#### Model fit and generating parameter values

The columns labeled “Estimated” in Table 1 show the estimated posterior means for our fit of Steingroever et al.’s data. As can be seen, the regression weights $\alpha _{z^{\prime }j}$ for the regression of participants’ model parameters on their covariate values are relatively small; the strongest relationships are between the outcome sensitivity parameter *A* and the Deliberation scale, and between the loss aversion parameter *w* and the Intuition scale (i.e., $\alpha _{A^{\prime }1}=0.61$ and $\alpha _{w^{\prime }2}=-0.51$, respectively). The corresponding Bayes factors are shown in the columns labeled “ BF_10*RG*_”. As can be seen, the Bayes factors for the relationship between outcome sensitivity *A* and the Deliberation scale *x*_1_, and between loss aversion *w* and the Intuition scale *x*_2_, are relatively modest, with values of 7 and 6.65, respectively. Moreover, the Bayes factors for the remaining relationships between model parameters and covariates are close to one, indicating that the data are nondiagnostic. In light of the sample size in Steingroever et al.’s study and our a priori expectation of relatively small effects sizes, these small Bayes factors suggest that applications of our regression framework require a more sizable data set to obtain substantial evidence for relationships between model parameters and covariates.

**Table 1:** Posterior estimates of parameter values for Steingroever et al.’s (in press) data, adjusted parameter values used to generate synthetic data, and Bayes factors for Steingroever et al.’s (in press) data

	Estimated				Adjusted				BF_10*RG*_		BF_10*MS*_	
*z* ^′^	$\alpha _{z^{\prime }1}$	$\alpha _{z^{\prime }2}$	$\mu _{z^{\prime }}$	$\sigma _{z^{\prime }}$	$\alpha _{z^{\prime }1}$	$\alpha _{z^{\prime }2}$	$\mu _{z^{\prime }}$	$\sigma _{z^{\prime }}$	$x_{1}$	*x*_2_	*x*_1_	*x*_2_
*A* ^′^	0.61	0.31	0.24	2.82	**1**	**0**	0.24	**1.06**	7.00	2.55	0.28	0.28
*w* ^′^	−0.04	−0.51	0.38	2.42	**0**	**−0.9**	0.38	**0.91**	1.44	6.65	0.27	0.27
*a* ^′^	−0.08	0.24	0.30	1.58	−0.08	0.24	0.30	1.58	0.98	2.07	0.27	0.26
*c* ^′^	−0.02	−0.05	1.34	0.46	−0.02	−0.05	1.34	0.46	0.27	0.39	0.26	0.26

Subscript 1 indicates effect sizes for the Deliberation scale, subscript 2 indicates effect sizes for the Intuition scale. *x*_1_ denotes the Deliberation scale, *x*_2_ denotes the Intuition scale. Bold parameter values were adjusted before generating synthetic data. BF_10*RG*_ is the Bayes factor for the regression analysis, and BF_10*MS*_ is the Bayes factor for the median-split analysis

The columns labeled “ BF_10*MS*_” in Table 1 show Bayes factors we obtained in a dichotomization-based analysis similar to that used by Steingroever et al. (in press; see section “Analysis Using Dichotomization” for details on this analysis). All Bayes factors from this analysis were close to 1. These results suggest that a dichotomization-based analysis requires an large data set and substantial effect sizes to be able to detect relationships between model parameters and covariates.

To be able to demonstrate the adverse effects of dichotomizing covariates, we needed to generate data with clearly identifiable relationships between model parameters and covariates (recall that, in the case of uncorrelated covariates, dichotomizing covariates should result in statistical tests missing existent effects). We therefore used twice the sample size of Steingroever et al.’s study for our simulations. Moreover, we set $\alpha _{A^{\prime }1}=1$ and $\alpha _{A^{\prime }2}=0$, which means that outcome sensitivity should be associated with deliberation but not intuition, and $\alpha _{w^{\prime }1}=0$ and $\alpha _{w^{\prime }2}=-0.9$, which means that loss aversion should be negatively associated with intuition but not deliberation. Because the regression weights $\alpha _{A^{\prime }.}$ and $\alpha _{w^{\prime }.}$ were now larger than the values estimated in our model fit, we needed to reduce the residual variance for the corresponding model parameters to maintain reasonable variance in the covariate scores between participants (compare Eq. 16). We therefore set the residual variances ${\sigma ^{2}_{A}}$ and ${\sigma ^{2}_{w}}$ to 3/8 the values estimated in our model fit. The resulting parameter values used to generate data in our simulations are shown in the columns labeled “Adjusted” in Table 1.

#### Data generation

For our simulations we generated 50 synthetic data sets under two different scenarios, one in which covariates were correlated and one in which covariates were uncorrelated. Each simulated data set consisted of 150 synthetic participants, which should allow our regression analysis to reliably detect relationships between model parameters and covariates. For each participant we generated two covariate values, *x*_1*i*_ and *x*_2*i*_, as well as one value for each of the four PVL-Delta model parameters. To obtain covariate values that were related to a specific model parameter but not to others, we started by generating a 2 × 1 vector of covariate values for each participant from a multivariate normal distribution, $\pmb {x}_{i} \sim \pmb {\mathscr {N}}(\pmb {\mu }, {\Sigma })$, with 2 × 1 mean vector *μ* =0, and 2 × 2 covariance matrix Σ. In the scenario with uncorrelated covariates, the covariance matrix was the identity matrix. In the scenario with correlated covariates the covariance matrix had diagonal entries 1 and off-diagonal entries 0.7. In a second step, we generated probit-transformed PVL-Delta parameters for each participant using Eq. 15, $z^{\prime }_{i} \sim \mathscr {N}(\mu _{z^{\prime }} + \pmb {x}^{\prime T}_{i} \pmb {\alpha }_{z^{\prime }}, \sigma _{z^{\prime }})$. We set the data-generating group-level parameter values for the regression weights $\alpha _{z^{\prime }.}$, mean group-level parameters $\mu _{z^{\prime }}$, and residual variances $\sigma ^{2}_{z^{\prime }}$ to the values given in Table 1.

Finally, based on the four PVL-Delta parameters, we simulated 200 trials of the IGT for each participant. We doubled the number of trials per participant compared to the data in Steingroever et al. (in press) to reduce the impact of imprecise estimates of the PVL-Delta parameters on the estimation of the regression weights. To generate simulated IGT trials for each participant, we first spawned a set of payoffs for each deck of cards based on the payoff scheme used in Steingroever et al.’s (in press) study. We then applied the cumulative distribution function of the standard normal distribution to the probit-scaled model parameters *z* ^′^ generated previously to obtain the corresponding PVL-Delta parameter *z*. We furthermore initialized the expected utilities for all four decks of cards to 0, meaning that the choice of the first card was entirely random for all simulated participants. After generating a random choice on the first trial for each participant, we evaluated the outcome, updated the expected utilities, and generated the participant’s choice on the next trial using Eqs. 1–3 and the parameter values for that simulated participant. We continued this process iteratively until we had accumulated 200 simulated choices. Further details and the R code used to generate the simulated data can be found at osf.io/6tfz3.

### Analysis using dichotomized covariates

Dichotomization-based analysis strategies take several forms. One that is frequently seen in practice is the median-split. To emulate this analysis strategy in the context of our simulation study, we developed a version of the hierarchical Bayesian PVL-Delta model that estimates separate group-level means $\mu _{z^{\prime }}$ for participants scoring above versus below the median on a covariate. Note that including these separate group-level means in the model constitutes a relatively sophisticated version of a dichotomization-based analysis; in practice, researchers are more likely to engage in a two-step analysis approach, first fitting the cognitive model separately to the groups of participants scoring above versus below the median, and subsequently testing the estimated model parameters for differences between groups. However, such a two-step procedure introduces additional biases beyond those introduced by dichotomization which is beyond the scope of the present work (see Boehm et al., 2016, for a discussion).

Our median-split model assumes the same hierarchical structure as the PVL-Delta model, with trials nested within participants whose probit-transformed parameter values are sampled from a group-level normal distribution. The mean of the group-level distribution from which a participant’s probit-transformed parameter values are drawn depends on the person’s values on the covariates. We implemented this constraint using effect coding (Rouder et al. 2012), that is, we assigned each participant *i* a *P* × 1 vector *d*_*i*_ = [*d*_*i*1_,…,*d*_*iP*_] where the *j* th entry of the vector is 0.5 if the person’s score on covariate *j* is greater than the median, and -0.5 otherwise:

20 $$ z^{\prime}_{i} \sim \mathscr{N}(\mu_{z^{\prime}}+\pmb{\delta}^{T}_{z^{\prime}} \pmb{d}_{i} \sigma_{{z}^{\prime}},\sigma_{{z}^{\prime}}^{{2}}).  $$

Here $\mu _{z^{\prime }}$ is the mean of model parameter *z* ^′^. Furthermore, $\pmb {\delta }_{z^{\prime }}$ is the *P* × 1 vector of standardized effect sizes (i.e., $\pmb {\delta }_{z^{\prime }} = [\delta _{z^{\prime }1}, \hdots , \delta _{z^{\prime }P}]^{\prime }$) and $\delta _{z^{\prime }j}$ is the standardized effect size indicating the difference, in standard deviations, between participants with below-median values on covariate *j* and participants with an above-median value on covariate *j*. Finally, $\sigma ^{2}_{z^{\prime }}$ is the variance of the model parameter *z* ^′^ across participants.

As in the PVL-Delta model with the regression extension, we assigned the group-level means $\mu _{z^{\prime }}$ a standard normal prior $\mu _{z^{\prime }} \sim \mathscr {N}(0,1)$, and the group-level variance $\sigma ^{2}_{z^{\prime }}$ an inverse-gamma prior $\sigma ^{2}_{z^{\prime }} \sim \mathscr {I}\mathscr {G}(2,1/2)$ with shape parameter 2 and scale parameter 1/2. Finally, we assigned the standardized effect sizes $\delta _{z^{\prime }j}$ independent Cauchy priors $\delta _{z^{\prime }j} \sim \mathscr {C}(1)$ with scale parameter 1.

### Data analysis

We analyzed the simulated data using the PVL-Delta model with regression extension and the PVL-Delta model with the median-split. For both models we computed Bayes factors contrasting the null hypothesis that there is no relationship between model parameters and covariates with the alternative hypothesis that there is such a relationship. More specifically, in the case of the regression model, the null hypothesis stated that the standardized effect size for a specific model parameter *z* ^′^ on a specific covariate *x*_*j*_ is 0, $\mathscr {H}_{0}: \beta _{z^{\prime }j}=0$, and the alternative hypothesis stated that the standardized effect size is not 0, $\mathscr {H}_{1}: \beta _{z^{\prime }j} \sim \mathscr {C}(1)$. As we expected sizable effects in the simulated data, we set the scale parameter for the regression model’s Cauchy prior to *s* = 1 (as in Rouder et al., 2009; Jeffreys, 1961). In the case of the median-split model, the null hypothesis stated that the standardized difference in group means is 0, $\mathscr {H}_{0}: \delta _{z^{\prime }j}=0$, and the alternative hypothesis stated that the difference in group means is not 0, $\mathscr {H}_{1}: \delta _{z^{\prime }j} \sim \mathscr {C}(1)$.

We based our computation of the Bayes factors for both models on the Savage-Dickey density ratio (Dickey & Lients 1970; Wagenmakers et al., 2010). To obtain estimates of the posterior density for each model’s effect size parameters, we first implemented both models in Stan (Carpenter et al., in press; Stan Development Team, 2016a, b). We subsequently ran two MCMC chains for each model parameter and collected 45,000 posterior samples per chain. We discarded the first 5,000 samples of each chain as burn-in and furthermore thinned each chain, retaining every fifth sample, which left us with a total of 8,000 samples per chain. All chains showed good convergence (Gelman-Rubin diagnostic $\hat {R} \leq 1.001$; Gelman & Rubin, 1992). We estimated the density of the posteriors for the $\beta _{z^{\prime }j}$ and $\delta _{z^{\prime }j}$ using log-spline functions, and computed the Bayes factors by taking the ratio of posterior densities to the prior densities at 0.

### Results

**Fig. 3:**
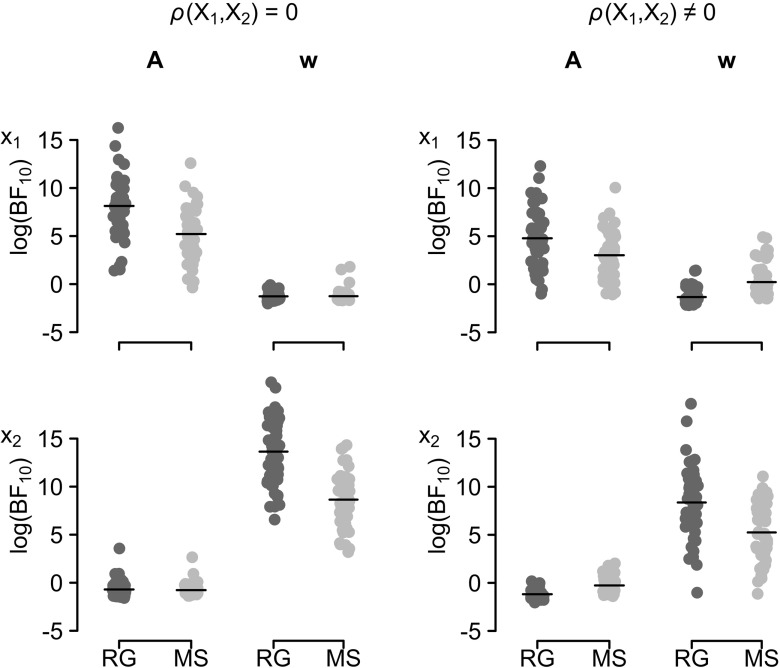
Bayes factors from 50 simulated data sets for the regression and median-split analysis. Data points show the log-Bayes factors for the alternative hypothesis (log(BF_10_)) obtained in the regression (RG, *dark gray dots*) and median-split (MS, *light gray dots*) analysis for the PVL-Delta model’s *A* and *w* parameters (*columns*) and two covariates (*rows*). The left subplot shows the results for the case of uncorrelated covariates, the right subplot shows the results for the case of correlated covariates. Lines indicate the mean log-BF. Data points are jittered along the x-axis for improved visibility

Figure 3 shows the log-Bayes factors for the alternative hypothesis obtained in our simulations. We chose to plot the log of the Bayes factors here, rather than the Bayes factors, because the Bayes factors spanned up to five orders of magnitude, which means that, on the linear scale, large Bayes factors would obscure differences in Bayes factors at the low end of the scale. Moreover, because we generated our data in such a way that only the PVL-Delta parameters *A* and *w* had sizable relationships with the covariates, we will only present the results for these parameters here. The full results for all model parameters as well as details on the estimated effect sizes can be found in the Appendix. The left panel of Fig. 3 shows the log-Bayes factors for our simulations with uncorrelated covariates. As can be seen, the Bayes factors obtained from the regression analysis showed strong evidence for an effect of the first covariate on the *A* parameter (dark gray dots, left column in the top row), whereas the median-split analysis provided much weaker evidence for such an effect (light gray dots, left column in the top row). This is also reflected in the median difference in log-Bayes factors, log(BF_10*RG*_) − log(BF_10*MS*_), of 2.82. On the linear scale this corresponds to regression Bayes factors being about 17 times the size of median-split Bayes factors, which indicates a strong underestimation of the evidence in the median-split analysis. Similarly, the regression analysis strongly supported an effect of the second covariate on the *w* parameter (dark gray dots, right column in the bottom row), whereas the median-split analysis provided much weaker evidence for such an effect (light gray dots, right column in the bottom row). The mean difference in log-Bayes factors was 13.70, which corresponds to regression Bayes factors being 890,537 times the size of median-split Bayes factors, indicating a tremendous underestimation of the evidence in the median-split analysis. For the null-effects of the first covariate on the *w* parameter (right column, top row) and of the second covariate on the *A* parameter (left column, bottom panel), both analyses performed similarly, with median differences in log-Bayes factors of 0.06 and 0.09, respectively. This corresponds to ratios of regression to median-split Bayes factors on the linear scale of 1.07 and 1.09, respectively, indicating only negligible differences.

The right panel for Fig. 3 shows the log-Bayes factors for our simulations with correlated covariates. The Bayes factors obtained from the regression analysis again showed stronger evidence for an effect of the first covariate on the *A* parameter (dark gray dots, left column in the top row) than the median-split analysis (light gray dots, left column in the top row). However, the median difference in log-Bayes factors of 1.55, which corresponds to regression Bayes factors being about 5 times the size of median-split Bayes factors on the linear scale, was much smaller than in the case of uncorrelated covariates. Similarly, the regression analysis provided stronger support for an effect of the second covariate on the *w* parameter (dark gray dots, right column in the bottom row), than the median-split analysis (light gray dots, right column in the bottom row). This is also reflected in the median difference in log-Bayes factors of 3.68, or a ratio of regression Bayes factors to median-split Bayes factors of 40 on the linear scale, which is still sizable but smaller than in the case of uncorrelated covariates. Unlike in the case of uncorrelated covariates, in the case of correlated covariates the median-split analysis now suggested a spurious effect of the first covariate on the *w* parameter (right column, top row), with a median difference in log-Bayes factors of −1.28, which corresponds to a ratio of median-split Bayes factors to regression Bayes factors of about 4 on the linear scale. Here, the negative sign of the median difference in log-Bayes factors indicates that the regression analysis tended to favor the null hypothesis whereas the median-split analysis favored the alternative hypothesis. Moreover, the median-split analysis also suggested a spurious effect of the second covariate on the *A* parameter, with a median difference of −0.68, which corresponds to a ratio of median-split Bayes factors to regression Bayes factors of about 2 on the linear scale.

**Fig. 4:**
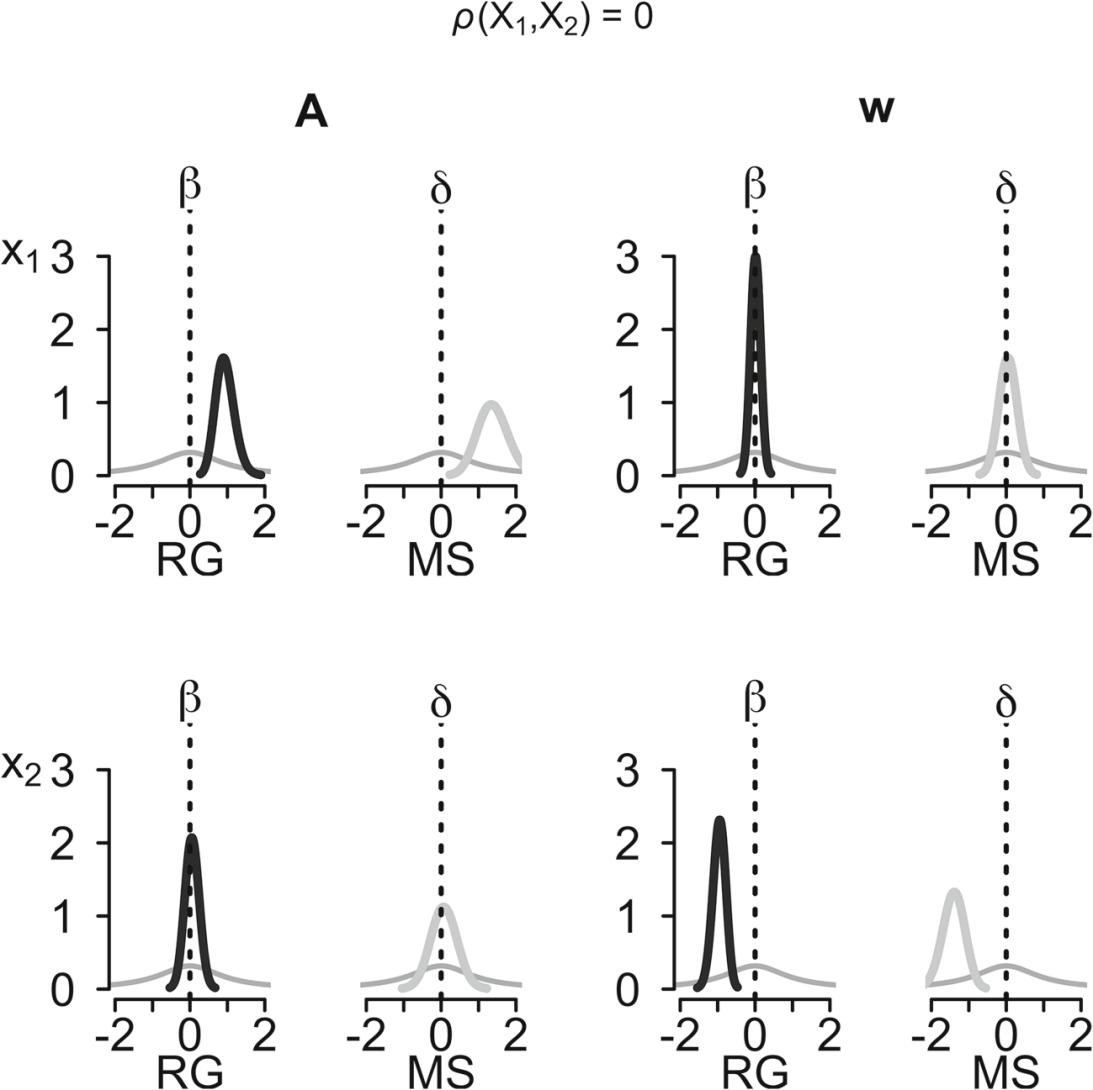
Posterior distributions of effect sizes for the case of uncorrelated covariates. Shown are the posterior distributions quantile-averaged across 50 simulated data sets. The left subplot shows the results for the *A* parameter, the right subplot shows the results for the *w* parameter. *Thick black lines* are the posteriors of the standardized effect sizes *β* (left column in each subplot), *thick gray lines* are the posteriors of the standardized mean differences *δ* (right column in each subplot), *thin gray lines* show the priors. The *top row* shows the results for the first covariate (*X*_1_), the *bottom row* shows the results for the second covariate (*X*_2_)

The biases inherent in the median-split analysis are also clearly visible in the posterior distributions for the effect sizes. Figure 4 shows the posterior distributions of the standardized differences in group means, *δ*, and the standardized effect sizes, *β*, quantile-averaged across simulations, for the case of uncorrelated covariates. The left column of the top left subplot shows the prior (thin gray line) and the posterior (thick black line) for the regression of the *A* parameter on the first covariate. Compared to the prior, which has considerable mass at the point null hypothesis *β*_*A*,1_ = 0, the posterior has nearly no mass at the point null, resulting in Bayes factors that strongly favor the alternative hypothesis. The right column in the same subplot shows the prior (thin gray line) and posterior (thick gray line) for the standardized difference in the *A* parameter between participants who score above-median on the first covariate and participants who score below-median. As can be seen, the posterior has little mass at the point null hypothesis *δ*_*A*,1_ = 0, resulting in Bayes factors favoring the alternative hypothesis. However, compared to the posterior under the regression model, the posterior under the median-split model is considerably wider and has more mass at the point null, which results in the underestimation of the evidence against the null observed above. A comparable pattern can be seen in the bottom right subplot; the posterior under the median-split model has more mass at the point null than the posterior under the regression model, resulting in a strong underestimation of the evidence against the null. Finally, the top right and bottom left subplots show the comparison for the true null-effects of the first covariate on the *A* parameter and of the second covariate on the *w* parameter, respectively. Although the posterior under the median-split model again has less mass at the point null than the posterior under the regression model, the differences are less pronounced and both models favor the null hypothesis.

**Fig. 5:**
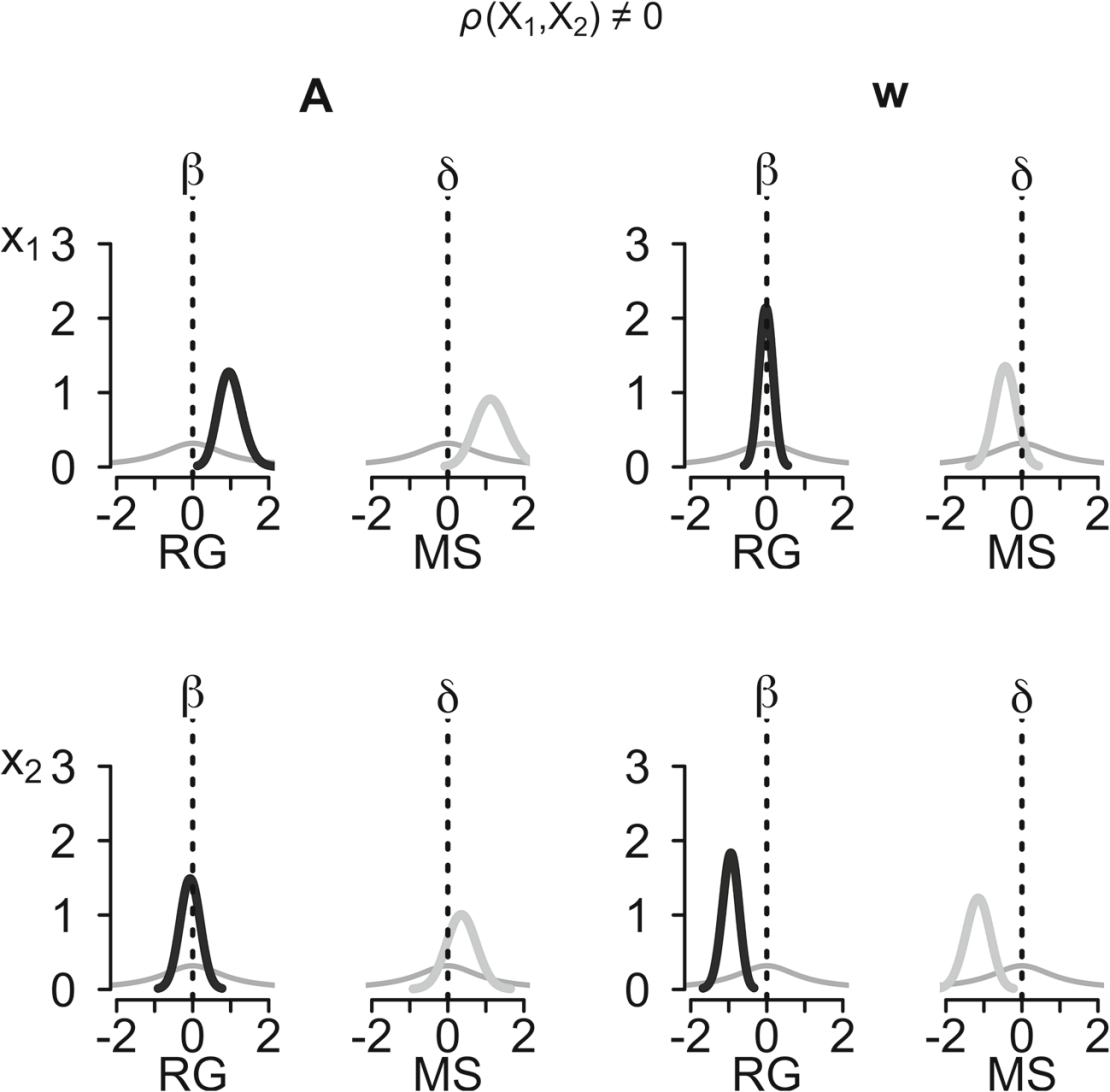
Posteriors of effect sizes for the case of correlated covariates. Shown are the posterior distributions quantile-averaged across 50 simulated data sets. The *left subplot* shows the results for the *A* parameter, the *right subplot* shows the results for the *w* parameter. *Thick black lines* are the posteriors of the standardized effect size *β* (*left column* in each subplot), *thick gray lines* are the posteriors of the standardized mean differences *δ* (*right column* in each subplot), *thin gray lines* show the priors. The *top row* shows the results for the first covariate (*X*_1_), the *bottom row* shows the results for the second covariate (*X*_2_)

Figure 5 shows the quantile-averaged posterior distributions of the standardized differences in group means, *δ*, and the standardized effect sizes, *β*, for the case of correlated covariates. The top left and bottom right subplots show comparable patterns to the case of uncorrelated covariates; the posterior under the regression and the median-split model both have much less mass at the point null than the respective prior, resulting in Bayes factors favoring the null hypothesis. However, compared to the prior, the posterior under the regression model is much narrower than the posterior under the median-split model, which leads to smaller Bayes factors under the median-split model. Finally, the top right and bottom left subplots show the comparison for the true null-effects of the first covariate on the *w* parameter and of the second covariate on the *A* parameter, respectively. As can be seen, the posterior for the regression weights is centered at 0 and has considerably more mass at the point null than the prior. Therefore, Bayes factors under the regression model correctly favor the null hypothesis. However, the posterior under the median-split model lies to the right of the point null for the *A* parameter and to the left of the point null for the *w* parameter, and thus has considerably less mass at the point null than the posterior under the regression model. Consequently, Bayes factors under the median-split model understate the evidence for the null and in many instances even support the alternative hypothesis, suggesting spurious associations between the first covariate and the *w* parameter and between the second covariate and the *A* parameter.

### Discussion

The goal of the present work was to develop a methodological framework that allows researchers to test hypotheses about associations between the parameters of a cognitive model and covariates in a principled way. To this end we showed how Bayesian linear regression can be used to obtain Bayes factors for specific associations between model parameters and covariates. As an example application, we chose the PVL-Delta model which aims to explain risky decision-making as the result of a reinforcement-learning process. Adding a regression extension to the PVL-Delta model allowed us to simultaneously account for participants’ model parameters and measurements of participants’ preferred decision styles.

One analysis strategy that has been used regularly to test for associations between model parameters and covariates is to divide participants into groups based on their covariate scores and subsequently test for differences in model parameters between groups of participants. Despite repeated warnings against the use of dichotomization-based analyses (Austin & Brunner 2004; Cohen 1983; MacCallum et al. 2002; Maxwell & Delaney 1993; Royston et al. 2006), a number of recent studies have relied on median-splits (e.g., Beitz et al., 2014; Cooper et al., 2015; Kwak et al., 2014; Steingroever et al., in press) to test for associations between the parameters of different cognitive models and covariates. We conducted a simulation study to illustrate the degree of bias introduced by such suboptimal analysis strategies. To this end, we generated simulated data under two scenarios. In one scenario covariates were not correlated with each other, and some of the covariates were correlated with some of the model parameters but not others. In the other scenario covariates were correlated with each other, and some of the covariates were correlated with some of the model parameters but not others. Our simulations showed that, in the first scenario, a median-split analysis leads to Bayes factors that understate the evidence for true effects compared to the Bayes factors obtained from a regression model. In the second scenario, Bayes factors from a median-split analysis again understated the evidence for true effects but, in addition, a median-split analysis also suggested spurious effects of covariates on model parameters that were, in fact, unrelated.

Interestingly, for the median-split analysis as well as the regression analysis, Bayes factors favoring the null hypothesis were very modest across simulations compared to Bayes factors favoring the alternative hypothesis. This is a well-known theoretical result that is due to different rates of convergence for Bayes factors under the two hypotheses. In particular, if data are generated under the alternative hypothesis, the rate of convergence of Bayes factors will be in the order of $\sqrt {n}$ whereas if data are generated under the null hypothesis, the rate of convergence will be in the order of log *n*, and thus much lower (Bahadur & Bickel 2009; Johnson 2010).

The reason for this different rate of convergence is that the alternative hypothesis is centered at the value of the point null hypothesis, and thus gives high plausibility also to data generated under the null hypothesis. Therefore, if data are generated under the null hypothesis, the null hypothesis gains evidential support over the alternative hypothesis only because it offers a more parsimonious account of the data. If the data are generated under the alternative hypothesis, on the other hand, the alternative quickly gains support over the null hypothesis because the data are highly implausible under the null hypothesis. Consequently, finding strong evidence for the null hypothesis will require considerably more data than finding evidence for the alternative hypothesis.

Another interesting results of our simulations was that the Bayes factors for spurious effects suggested by the median-split analysis were relatively small compared to the Bayes factors for true effects. This result is due to the fact that the median-split analysis generally leads to wider posteriors than the regression analysis, resulting in overall smaller Bayes factors.

Finally, the results of our present work imply that practical applications of our regression framework require a sufficiently large sample size. The application of our regression framework to Steingroever et al.’s (in press) data yielded relatively modest Bayes factors. To be able to obtain sizable Bayes factors in our simulations, we generated relatively large data sets. This suggests that researchers who wish to apply our regression framework will need to acquire sufficiently large data sets to be able to find substantial support for relationships between model parameters and covariates.

Our focus in the present work has been on the development of a regression framework for relating covariates to model parameters. An alternative approach to establishing relationships between model parameters and covariates is to estimate their joint covariance matrix. For example, Turner et al. (2013) have presented a hierarchical Bayesian joint modeling approach where covariates and parameters of a cognitive model are related via a joint covariance matrix. However, this approach comes with a number of practical challenges. Firstly, estimating models with highly correlated parameters often requires specialized sampling algorithms (e.g., Turner et al., 2013) that are not available in standard Bayesian software packages such as Stan (Carpenter et al., in press; Stan Development Team, 2016a, b) or JAGS (Plummer, 2003). The regression framework presented here, on the other hand, can easily be implemented using standard sampling algorithms for many types of cognitive models (see below). Secondly, many joint modeling implementations have been developed for the purpose of estimation rather than hypo- thesis testing (e.g., Turner et al., 2013; Michalkiewicz & Erdfelder, 2016; Vandekerckhove et al., 2011). As such, this work has not addressed the problem of how to select an appropriate prior distribution for the covariance matrix, which critically determines the properties of Bayesian statistical tests. The effect of different prior distributions for regression coefficients on Bayes factors, on the other hand, has been thoroughly investigated and prior distributions have been developed that satisfy a number of theoretical desiderata (e.g., Liang et al., 2008; Rouder & Morey, 2012; Zellner, 1986). Thirdly, a related problem concerns how associations between individual covariates and model parameters can be tested. While some work has addressed the problem of testing correlations between a single covariate and a specific model parameter (Matzke et al. 2017; Jeffreys 1961), it is not clear how to test individual entries from a covariance matrix if several covariates are included in a model simultaneously. The regression framework presented here, on the other hand, allows for straightforward tests of individual regression weights. Due to these practical limitations of the joint modeling approach we consider the regression approach presented here as the currently most practical solution to the problem of relating model parameters to covariates.

We have limited the application of our hierarchical Bayesian regression framework in the present work to the PVL-Delta model. However, the framework can also be adapted to other cognitive models that fulfill a few modest requirements. Firstly, the cognitive model needs to be implemented in a hierarchical way to allow researchers to relate individual participants’ model parameters to measured covariates. For many popular models such hierarchical implementations are readily available (Matzke et al., 2015; Steingroever et al., 2014; Wiecki et al., 2013; Ahn et al.’s, 2016, R-package contains hierarchical implementations of several popular models of decision-making) or can be easily developed using MCMC software packages such as JAGS (Plummer, 2003) or Stan (Carpenter et al., in press; Stan Development Team, 2016a.

Secondly, the model parameters of interest need to be normally distributed. Although this assumption is often reasonable and can be readily adopted, in other cases the cognitive interpretation of the parameters or mathematical constraints necessitate specific bounds on the parameter values. However, such constraints can often be overcome by using an appropriate transformation of the model parameters, rather than the model parameters themselves, in the regression analysis. In the case of the PVL-Delta model, for instance, all model parameters are assumed to be restricted to closed intervals, yet probit transforming the parameters allowed us to add the Bayesian regression extension to the PVL-Delta model. One slight drawback of such non-linear transformations of model parameters is that the regression weights themselves can no longer be interpreted. However, in most practical applications researchers are only interested in the direction of the relationship between model parameters and covariates, which is not affected by monotonic transformations.

Thirdly, model parameters must be assumed to be dependent on the covariates. Linear regression is based on the assumption that the predictor variables cause the criterion variable. As we treat the model parameters as the criterion variable in our regression framework, applications of the framework are limited to situations where it is reasonable to assume that the model parameters depend on the covariates. These three conditions are all that is required for our regression extension to be added to a cognitive model and are easily met by most existing models.

Although reinforcement learning models, and the PVL-Delta model in particular, served merely as an example for our Bayesian regression framework, we believe that our regression extension can greatly facilitate research involving risky decision-making. One potential application beyond identifying relationships between model parameters and physiological measurements is the statistical control of nuisance variables. A number of authors have suggested that performance on the IGT might be subject to practice effects (Ernst et al., 2003; Lejuez et al., 2003; Verdejo- García & Pírez-García, Verdejo-Garcia2007), although no study to date has comprehensively addressed this problem (Buelow & Suhr 2009). Including time-on-task as a covariate in model-based analyses might allow researchers not only to control for practice effects but also to pinpoint which cognitive processes are affected by practice and which processes remain stable over time. Similar model-based analyses in perceptual decision-making, for example, have suggested that while participants’ processing of stimuli remains unaffected by practice, their response mode can change over time although considerable practice might be needed for participants to reach optimal performance (Hawkins et al. 2015; Simen et al. 2009).

To conclude, in the present work we presented a hierarchical Bayesian regression extension for cognitive models that allows researchers to test for relationships between model parameters and covariates using Bayes factors. In our simulation study we showed how our regression framework overcomes the biases associated with the often-practiced median-split analysis. The latter can lead researchers to either miss existing relationships between model parameters and covariates, or suggest spurious associations between model parameters and covariates, depending on whether the covariates are correlated with each other or not. Moreover, compared to other methods for relating model parameters to covariates, such as joint modeling, our regression framework has relatively modest technical requirements and can be easily applied to many existing cognitive models. Consequently, our regression framework provides a practical and easy-to-use method for testing for relationships between model parameters and covariates.

## Figures and Tables

**Fig. 6:**
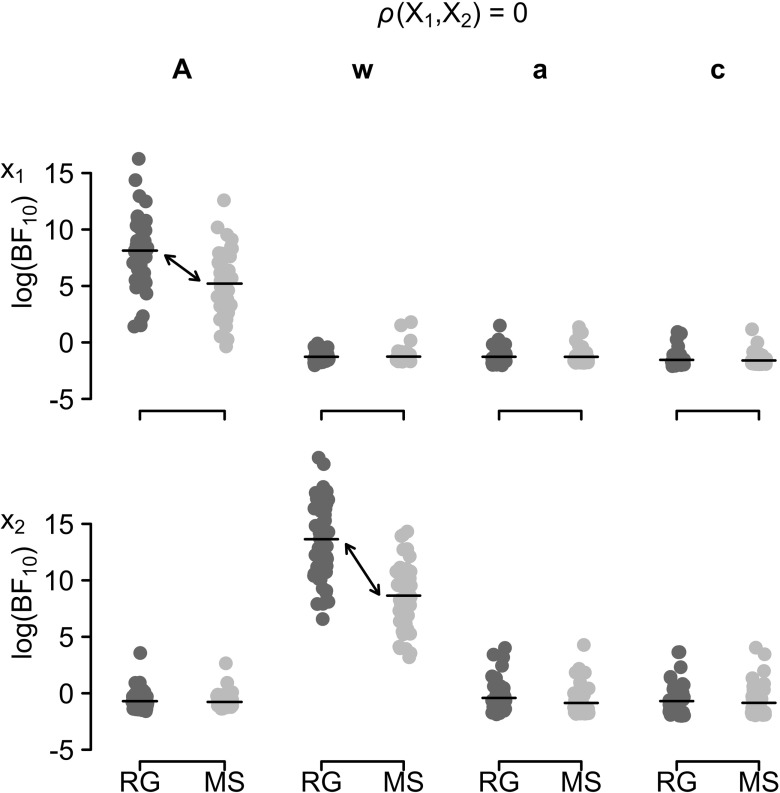
Bayes factors from 50 simulated data sets for the regression and median-split analysis with uncorrelated covariates. Data points show the log-Bayes factors for the alternative hypothesis (log(BF_10_)) obtained in the regression (RG, *dark gray dots*) and median-split (MS, *light gray dots*) analysis for the PVL-Delta model’s *A* and *w* parameters (*columns*) and two covariates (*rows*). *Lines* indicate the mean log-BF. *Arrows* highlight underestimation of Bayes factors in the median-split analysis. Data points are jittered along the x-axis for improved visibility

**Fig. 7:**
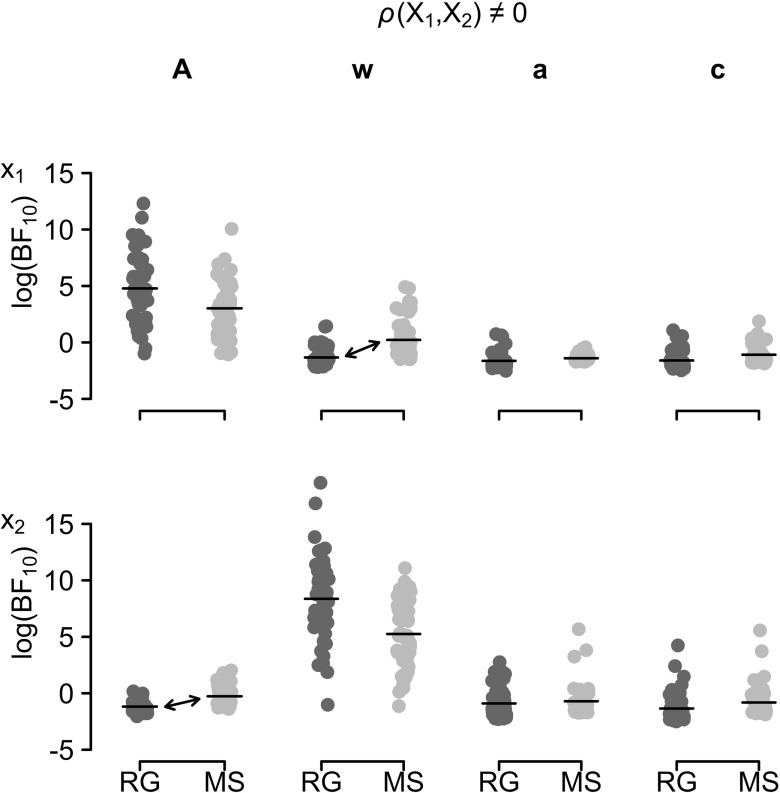
Bayes factors from 50 simulated data sets for the regression and median-split analysis with correlated covariates. Data points show the log-Bayes factors for the alternative hypothesis (log(BF_10_)) obtained in the regression (RG, *dark gray dots*) and median-split (MS, *light gray dots*) analysis for the PVL-Delta model’s *A* and *w* parameters (*columns*) and two covariates (*rows*). *Lines* indicate the mean log-BF. *Arrows* highlight overestimation of Bayes factors in the median-split analysis. Data points are jittered along the x-axis for improved visibility

**Fig. 8:**
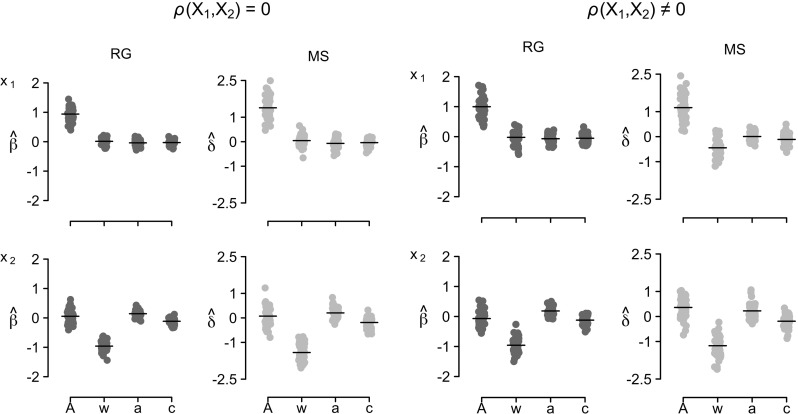
Mean posterior estimates from 50 simulated data sets of effects for the regression and median-split analysis for uncorrelated (*left subplot*) and correlated (*right subplot*) covariates. Data points show the estimated standardized effect sizes ($\hat {\beta }$) from the regression analysis (RG; left panels in each subplot, *dark gray dots*) and the estimated standardized effect size ($\hat {\delta }$) from the median-split analysis (MS; right panels in each subplot, *light gray dots*) for the four PVL-Delta parameters. The *top row* shows the results for the first covariate, the bottom row for the second covariate. *Black lines* indicate the mean across simulations. Data points are jittered along the x-axis for improved visibility.

## Appendix: Complete results of the simulation study

In this appendix we provide the results of our simulation study for all four parameters of the PVL-Delta model. Figure 6 gives the log-Bayes factors for all PVL-Delta parameters from our simulations with uncorrelated covariates. Dark gray dots show the Bayes factors obtained in the regression analysis, light gray dots show the Bayes factors obtained in the median-split analysis. Recall that our simulated data were generated so that the first covariate would be positively correlated with the *A* parameter and the second covariate would be negatively correlated with the *w* parameter. The correlations between *A* and the second covariate, and between *w* and the first covariate were set to 0 and the relationships between the remaining model parameters and the covariates were set to the values estimated from Steingroever et al.’s (in press) data, and were negligible. As described in the main text, the Bayes factors from the regression analysis showed strong evidence for an effect of the first covariate on the *A* parameter (dark gray dots, left column in the top row) whereas the median-split analysis provided much weaker evidence for such an effect (light gray dots, left column in the top row). Similarly, the regression analysis strongly supported an effect of the second covariate on the *w* parameter (dark gray dots, second column in the bottom row), whereas the median-split analysis provided weaker evidence for such an effect (light gray dots, second column in the bottom row). For the null-effects of the first covariate on the *w* parameter (second column, top row) and of the second covariate on the *A* parameter (left column, bottom panel), both analyses performed similarly without any appreciable differences in Bayes factors. Finally, both analyses provided only weak support if any for an effect the covariates on the *a* and *c* parameters and there were no sizeable differences in Bayes factors.

Figure 7 gives the log-Bayes factors for all PVL-Delta parameters from our simulations with correlated covariates. As described in the main text, the Bayes factors obtained from the regression analysis again showed stronger evidence for an effect of the first covariate on the *A* parameter (dark gray dots, left column in the top row) than the median-split analysis (light gray dots, left column in the top row). Similarly, the regression analysis provided stronger support for an effect of the second covariate on the *w* parameter (dark gray dots, second column in the bottom row), than the median-split analysis (light gray dots, second column in the bottom row). However, unlike in the case of uncorrelated covariates, in the case of correlated covariates the median-split analysis now suggested spurious effects of the first covariate on the *w* parameter (second column, top row) and of the second covariate on the *A* parameter (left column, bottom row). Finally, the regression as well as the median-split analysis did not provide strong evidence for any effects of the covariates on the *a* and *c* parameters, and there were no clear differences in Bayes factors visible between the two analyses.

Taken together, these results illustrate that, in the case of uncorrelated covariates, a median-split analysis tends to understate the evidence for existent effects. In the case of correlated covariates, a median-split analysis also understates the evidence for existent effects but in addition suggests spurious effects of covariates on model parameters that are in fact unrelated. Furthermore, our results show that in cases where model parameters do not have any appreciable relationships with any of the covariates, as was the case for the *a* and *c* parameters, regression and median-split analyses perform similarly and there are no appreciable biases associated with a dichotomization-based analysis.

Figure 8 shows the posterior means of the standardized effect sizes estimated in the regression analysis (RG, left panels in each subplot) and the posterior means of the standardized effect sizes estimated in the median-split analysis (MS, right panels in each subplot). The left subplot shows results for the case of uncorrelated covariates, the right subplot shows the results for the case of correlated covariates. The top row shows the results for the first covariate, the bottom row for the second covariate. The results corroborate the results from the Bayes factors. In the case of uncorrelated covariates (left subplot), the estimated effects in both models are largest for effects that we created to be non-zero (i.e., the effect of the first covariate on *A* and the effect of the second covariate on *w*, leftmost column of the panels in the top row and second-from-left column of the panels in the bottom row, respectively). Moreover, both models correctly estimate the direction of the effect of the first covariate on the *A* parameter to be positive (leftmost column of the panels in the top row), and the direction of the effect of the second covariate on *w* to be negative (second-from-left column of the panels in the bottom row). Both models also correctly estimate the effects of the first and second covariate on *a* and *c* to be close to 0 (second-from-right and rightmost columns of each panel).

In the case of correlated covariates (right subplot), both analyses again correctly estimate the size and direction of the strong effects of the first covariate on the *A* parameter (leftmost column of the panels in the top row) and of the second covariate on the *w* parameter (second-from-left column of the panels in the bottom row). However, while the regression analysis correctly estimates the relationships between the first covariate and the *w* parameter (left panel, second-from-left column in the top row) and between second covariate and the *A* parameter (left panel, leftmost column in the bottom row) to be approximately 0, the median-split analysis suggests a weakly negative association between the first covariate and *w* (right panel, second-from-left column in the top row) and between the second covariate and *A* (right panel, leftmost column in the bottom row). Finally, both models correctly estimate the effects of the covariates on the *a* and *c* parameters to be close to 0.

These results align well with the results for the Bayes factors. In the case of uncorrelated covariates, the regression analysis as well as the median-split analysis correctly indicate the direction and size of the relationships between covariates and model parameters. However, in the case of correlated covariates, the median-split analysis tends to suggest spurious relationships between covariates and model parameters. The direction of these spurious effects is equal to the direction of the true effects. This suggests a spill-over from one covariate to the other that arises from the fact that the median-split analysis ignores the correlation between covariates, whereas the regression analysis partials out correlations between covariates.

## Footnotes

1 However, these limitations do not apply to the Neymann-Pearson approach to hypothesis testing. In this approach, the researcher tests a specific alternative hypothesis against a null hypothesis. The choice of the significance threshold is based on a tradeoff between Type I and Type II errors, and sample size. If the p-value exceeds the significance threshold, the researcher acts as if the alternative hypothesis were true. If the p-value falls below the significance threshold, the researcher acts as if the null hypothesis were true (e.g., Gigerenzer et al., 2004).

2 Note that because the design matrix appears in the expression for the prior in the inverse of the matrix (*X* ^*T*^ *X*)^−1^, multiplying (*X* ^*T*^ *X*)^−1^ by *N* is equivalent to diving (*X* ^*T*^ *X*) by *N*.

3 However, performing the same analyses with the scale parameter set to *s* = 1, the default value, resulted in negligible changes in the posterior estimates of all parameters except *α*_*A*1_ and *α*_*A*2_. As we adjusted these parameters in the data generation step of our simulations (see section “Data generation”), our choice of the scale parameter did not influence the results of our simulations.
